# Stable and High Piezoelectric Output of GaN Nanowire-Based Lead-Free Piezoelectric Nanogenerator by Suppression of Internal Screening

**DOI:** 10.3390/nano8060437

**Published:** 2018-06-14

**Authors:** Muhammad Ali Johar, Mostafa Afifi Hassan, Aadil Waseem, Jun-Seok Ha, June Key Lee, Sang-Wan Ryu

**Affiliations:** 1 Department of Physics, Chonnam National University, Gwangju 61186, Korea; alijoharphysicst@gmail.com (M.A.J.); afifiphysics@gmail.com (M.A.H.); waseemphycnu@gmail.com (A.W.); 2 Optoelectronics Convergence Research Center, Chonnam National University, Gwangju 61186, Korea; jsha@jnu.ac.kr (J.-S.H.); junekey@chonnam.ac.kr (J.K.L.)

**Keywords:** GaN nanowires, nanogenerator, piezoelectricity, flexible electronics, free-carrier screening

## Abstract

A piezoelectric nanogenerator (PNG) that is based on c-axis GaN nanowires is fabricated on flexible substrate. In this regard, c-axis GaN nanowires were grown on GaN substrate using the vapor-liquid-solid (VLS) technique by metal organic chemical vapor deposition. Further, Polydimethylsiloxane (PDMS) was coated on nanowire-arrays then PDMS matrix embedded with GaN nanowire-arrays was transferred on Si-rubber substrate. The piezoelectric performance of nanowire-based flexible PNG was measured, while the device was actuated using a cyclic stretching-releasing agitation mechanism that was driven by a linear motor. The piezoelectric output was measured as a function of actuation frequency ranging from 1 Hz to 10 Hz and a linear tendency was observed for piezoelectric output current, while the output voltages remained constant. A maximum of piezoelectric open circuit voltages and short circuit current were measured 15.4 V and 85.6 nA, respectively. In order to evaluate the feasibility of our flexible PNG for real application, a long term stability test was performed for 20,000 cycles and the device performance was degraded by less than 18%. The underlying reason for the high piezoelectric output was attributed to the reduced free carriers inside nanowires due to surface Fermi-level pinning and insulating metal-dielectric-semiconductor interface, respectively; the former reduced the free carrier screening radially while latter reduced longitudinally. The flexibility and the high aspect ratio of GaN nanowire were the responsible factors for higher stability. Such higher piezoelectric output and the novel design make our device more promising for the diverse range of real applications.

## 1. Introduction

The emergence of portable/wearable electronic gadgets that may accompany human body day-in and day-out has attracted worldwide attention of researchers due to their potential for a variety of applications. Among these, a variety of potential energy sources, mechanical sources, such as vibrations [1,2], movement [3,4], and wind waves are the most abundant in the surrounding environment [5,6]. The piezoelectric generators (PGs) that convert mechanical energy to electrical energy are considered the most favorable energy harvesters to power-up microelectronics. PGs may provide clean and inexhaustible means to power-up portable/wearable electronics and health monitoring sensors [7,8,9].

Traditionally, ceramic materials were used to fabricate PGs, but their applications remain limited due to very low output power density, such as quartz for watches and electric cigarette lighters [10,11,12]. Thus, better candidates for the fabrication of PGs that exhibit high power density are studied as semiconductors, polymers, and organic nanostructures. Among them, semiconductors have several advantages due to well-developed fabrication techniques. Upon straining the semiconductors, piezoelectric charges can be generated only in the non-centrosymmetric crystal structures [13]. The desirable examples are the III-V and II-VI semiconductors, which exhibit non-centrosymmetry. This property can be observed in wurtzite and zincblende crystal structures; the former has three independent piezoelectric coefficients e_31_, e_33_, and e_15_ while the latter has only one independent piezoelectric coefficient e_14_. The strongest piezoelectric semiconductor materials are from wurtzite family of crystal structure such as GaN, ZnO, InN, and AlN. The piezoelectric effect of semiconductors was studied in early 1960s to develop acoustic electronics in Bell Telephone laboratories [14,15,16]. A boom took place in semiconductor-based piezoelectricity after the report of Prof. Wang et al. in 2006 [17]. The aligned ZnO nanowires (NWs) were deflected by the tip of atomic force microscope in contact mode with simultaneous measurement of piezoelectric charges by the same set up in Schottky contact configuration. The power density of semiconductor-based PGs was high enough to power-up micro-electronic devices.

The semiconductor-based PGs can be fabricated using thin film or quasi one dimensional (1D) nanostructure; both the structures carry their pros and cons simultaneously. The fabrication of semiconductor thin films is easy, but the piezoelectric output is relatively low along with low compliance values. On the other hand, the fabrication of quasi 1D nanostructure is difficult, but it is accompanied with several advantages. Semiconductor NWs are the best candidate to fabricate compact and efficient PGs [18], because the piezoelectric properties are enhanced for NW-based piezoelectric nanogenerators (PNGs). A major problem for semiconductor-based PGs is the screening of piezoelectric charges due to free carriers inside semiconductors; however, NWs can offer a chance to suppress the screening effect. The surface states of NWs bend the conduction and the valance band edges upward of n-type semiconductor, which results in depletion of NWs. The complete depletion of NWs can be achieved for the NWs having a diameter lower than 30 nm [19]. Thus, free carrier screening is suppressed in quasi 1D nanostructure due to their lower dimensions. Moreover, NWs can be deformed elastically without plastic deformation due to their high flexibility, high compliance value, and high resistance to fatigue. Such mechanical properties may extend the operational lifetime of NWs based PNGs.

Among wurtzite semiconductor materials, ZnO is the most researched material for piezoelectric applications. GaN has a slightly lower piezoelectric coefficient, but it is considered to be a superior material due to its biocompatibility, mechanical robustness. In order to harvest high piezoelectric output from a semiconductor PG, suppression of internal screening by rectifying contact is an essential requirement. Forming a p-n homojunction and heterojunction is an efficient route to harvest high piezoelectric output by suppressing the free carrier screening and junction screening [20,21,22,23,24]. Until now, most of the PNGs that are based on GaN NWs have been constructed using Schottky junction configuration, as in the case of resiscopy [25,26]. However, the performance of Schottky junction based PNGs degrades significantly due to high reverse leakage current originating from defects as metal-semiconductor interface. This problem is deleterious for high piezoelectric performance because most of piezoelectric output is harvested during reverse bias mode. Moreover, the Schottky barrier height is greatly influenced by the applied stress to the PGs [27]. In this regard, PNGs incorporating a metal-polymer-semiconductor interface is an interesting approach for high piezoelectric performance. Its role at metal-semiconductor interface is similar to that of a dielectric due to its insulating nature. The piezoelectric output is expected to increase by the suppression of internal screening at the heterojunction [28,29,30,31]. The enhancement of piezoelectric output is studied only for ZnO based PNGs using metal-dielectric-semiconductor (MDS) interface. 

In this study, we have fabricated the GaN NW-based PNG using Ni as metal contact. The effect of MDS interface was studied to enhance piezoelectric output using Polydimethylsiloxane (PDMS) as dielectric medium between Ni electrode and GaN NWs. In order to use PNGs for real applications, it is necessary to fabricate them on flexible substrate [32,33,34,35]. The piezoelectric performance of PNG was measured, while the device was actuated by a cyclic stretching-releasing agitation mechanism driven by a linear motor. Moreover, the effect of actuation frequency on the piezoelectric performance was also evaluated. At the end, the long-term stability was checked for a real application of the device. A maximum piezoelectric output voltages and current were found 15 V and 85 nA, respectively. The primary responsible factors for very high piezoelectric output were surface Fermi level pinning and the insulating metal-dielectric-semiconductor interface due to the insertion of PDMS between metal and semiconductor. Our device exhibited very high piezoelectric output alongwith long-term stability, therefore the MDS interface can be used to fabricate semiconductor based PNGs for a variety of applications. 

## 2. Experimental Procedure

### 2.1. Growth of GaN Thin Film and Vertical GaN NWs

In order to demonstrate piezoelectricity from GaN NWs, only c-axis GaN NWs are feasible due to non-centrosymmetric behavior of crystal structure upon straining. In order to eliminate the stresses inside NWs due to lattice mismatch of NWs and substrate, 3 µm thick c-axis GaN thin film was grown on c-plane sapphire substrate. The flow rates of trimethylgallium (TMGa) and NH_3_ were 300 µmol/min and 450 mmol/min, respectively, which were the respective precursors for gallium and nitrogen; moreover the V/III ratio was kept constant at 1500. The growth was carried out at 1175 °C at the reactor pressure of 200 torr for 5500 s. In order to reduce the stress in GaN thin film due to lattice mismatch of substrate and the grown thin film of GaN, a low temperature buffer layer of GaN, was also grown at 600 °C. 

In order to grow c-axis GaN NWs, vapor-liquid-solid (VLS) technique was adopted. VLS technique requires a metal catalyst. Thus, 0.8 nm thick Au thin film was deposited by e-beam evaporation at vacuum of 5 × 10^−6^ torr on GaN thin film. Then, the samples were loaded inside the reactor of MOCVD. Then, the samples were annealed at 880 °C for 500 s in H_2_ environment, so that the agglomeration can take place. After the agglomeration of the catalyst, the precursors of gallium and nitrogen were introduced. TMGa and NH_3_ were used as precursors of gallium and nitrogen, respectively. The flow rate of TMGa and NH_3_ was 39.5 µmol/min and 6.7 mmol/min, respectively, with V/III ratio of 170. The growth of GaN NWs under VLS mode was carried out for 5000 s at 850 °C at the reactor pressure of 60 torr.

### 2.2. Fabrication of PNG

After the growth of high aspect ratio c-axis GaN NWs, PDMS was deposited by spin coating on GaN NWs and NWs embedded in PDMS is called matrix of NW-arrays. The spin coating was performed at 7000 rpm for 300 s. To harden PDMS, the samples were baked at 150 °C for 600 s. In order to complete the fabrication of PNG, ITO was deposited on Si-rubber substrate using sputtering. Then, the PDMS matrix embedded with GaN NW was cut using doctor blade, flipped and transferred on ITO coated Si-rubber substrate. Subsequently, PDMS was deposited to sandwich the matrix between PDMS and ITO-coated Si rubber. Then, Ni metal was deposited, followed by ITO deposition by sputtering to fabricate the top electrode of PNG.

### 2.3. Characterization of Structure

In order to evaluate the morphology of GaN NWs, field emission scanning electron microscope (JSM-6700 JEOL, Tokyo, Japan) was used. After the successful fabrication of PNGs, the piezoelectric performance of PNGs was measured by a high speed current-voltage measurement unit (PARSTAT 3000/Potentiostat/Galvanostat/ELS analyzer, Princeton Applied Research, AMETEK scientific instrument, Berwyn, PA, USA), while PNGs were actuated by a periodic stretching-releasing agitation driven by a linear motor.

## 3. Results and Discussions

**Figure 1:**
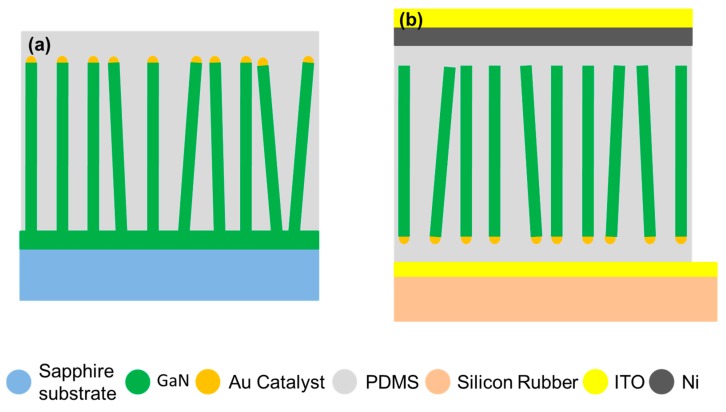
Schematic illustration of GaN NWs based piezoelectric nanogenerator (PNG) (**a**) c-axis GaN NWs after polydimethylsiloxane (PDMS) deposition on sapphire substrate; and, (**b**) transfer of PDMS matrix embedded with c-axis GaN NWs on Si-rubber substrate, NiO deposition and ITO electrode formation on complete device.

**Figure 2:**
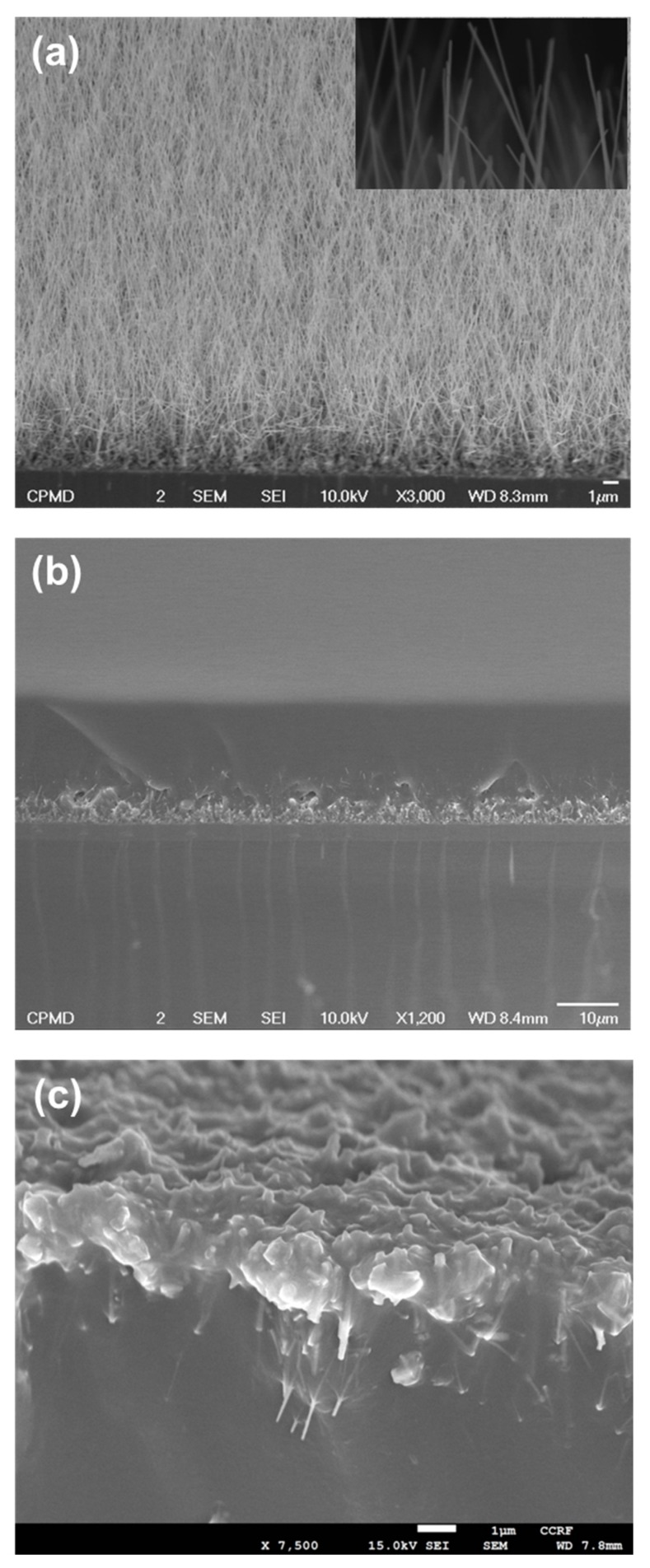
Scanning electron micrographs (**a**) vertical GaN NW on GaN thin film, inset shows the high magnification micrograph; (**b**) after deposition of PDMS on GaN NWs; and, (**c**) peeled-off PDMS matrix embedded with GaN NWs, bottom side of NWs is exposed while tope side is covered with PDMS.

In the case of semiconductors, the piezoelectric effect can be observed only from the crystal structures, which exhibit non-centrosymmetry upon straining. The wurtzite crystal structure is non-centrosymmetric along c-axis only. In this regard, c-axis GaN NWs were grown by the VLS mode using Au as catalyst. Due to the c-axis orientation of GaN thin film, GaN NWs were grown well aligned as depicted from the schematic illustration in Figure 1a. After the growth of c-axis GaN NWs, PDMS was deposited on GaN NW-arrays using spin coating. Then, the matrix of PDMS embedded with c-axis GaN NWs was transferred on silicon rubber substrate, followed by Ni deposition, and then top ITO electrode was formed by sputtering. The morphology of c-axis GaN NWs grown on c-axis GaN thin film is shown in Figure 2a. The diameter and length of GaN NWs were 54 nm and 10.5 µm, respectively, exhibiting a very high aspect ratio of 194. Moreover, inset shows a magnified view of NWs. Figure 2b shows the structure after deposition of PDMS, and it is very clear that NWs were embedded inside PDMS. The PDMS matrix embedded with c-axis GaN NWs was cut by doctor’s blading and its cross section is shown in Figure 2c after flipping.

**Figure 3:**
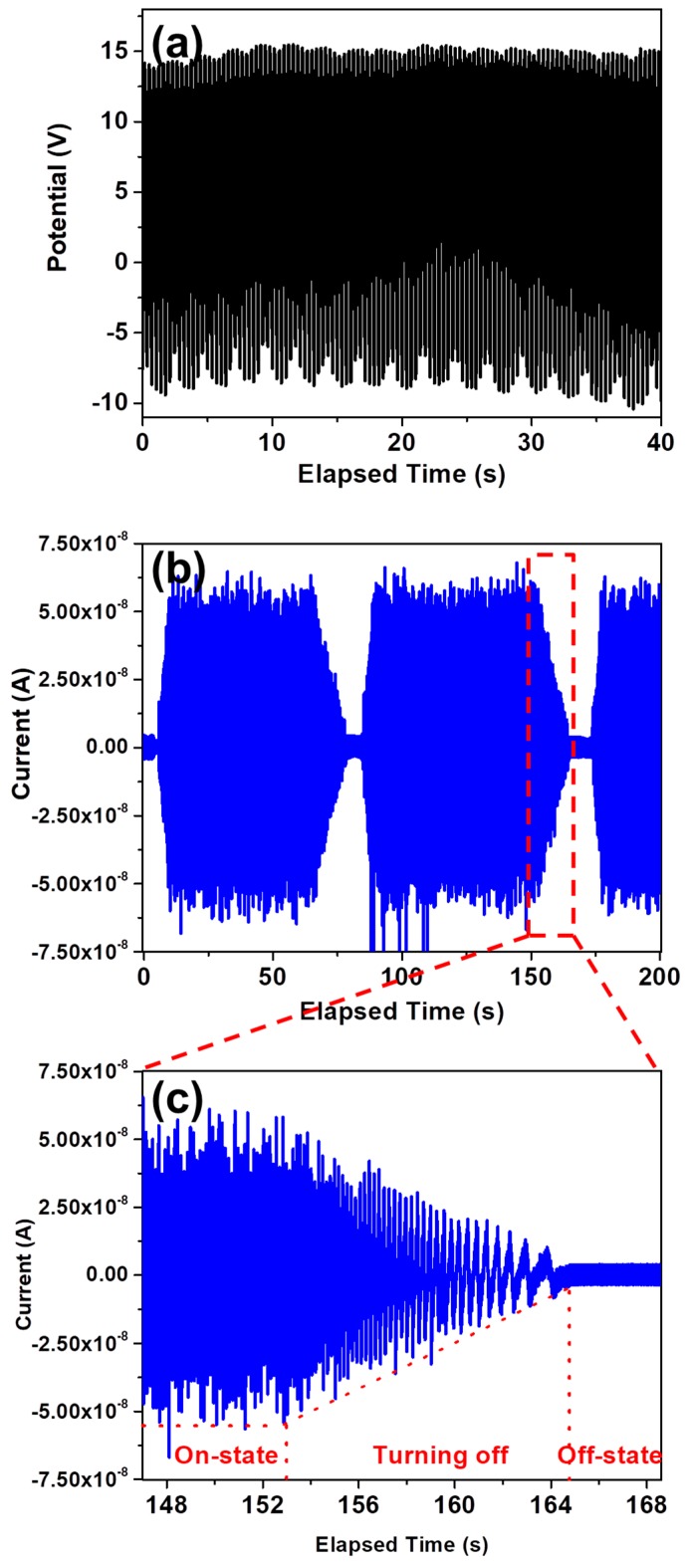
Piezoelectric outputs of PNG (**a**) open circuit voltages; (**b**) short circuit current; and, (**c**) magnified view of output current with decreasing frequency.

**Figure 4:**
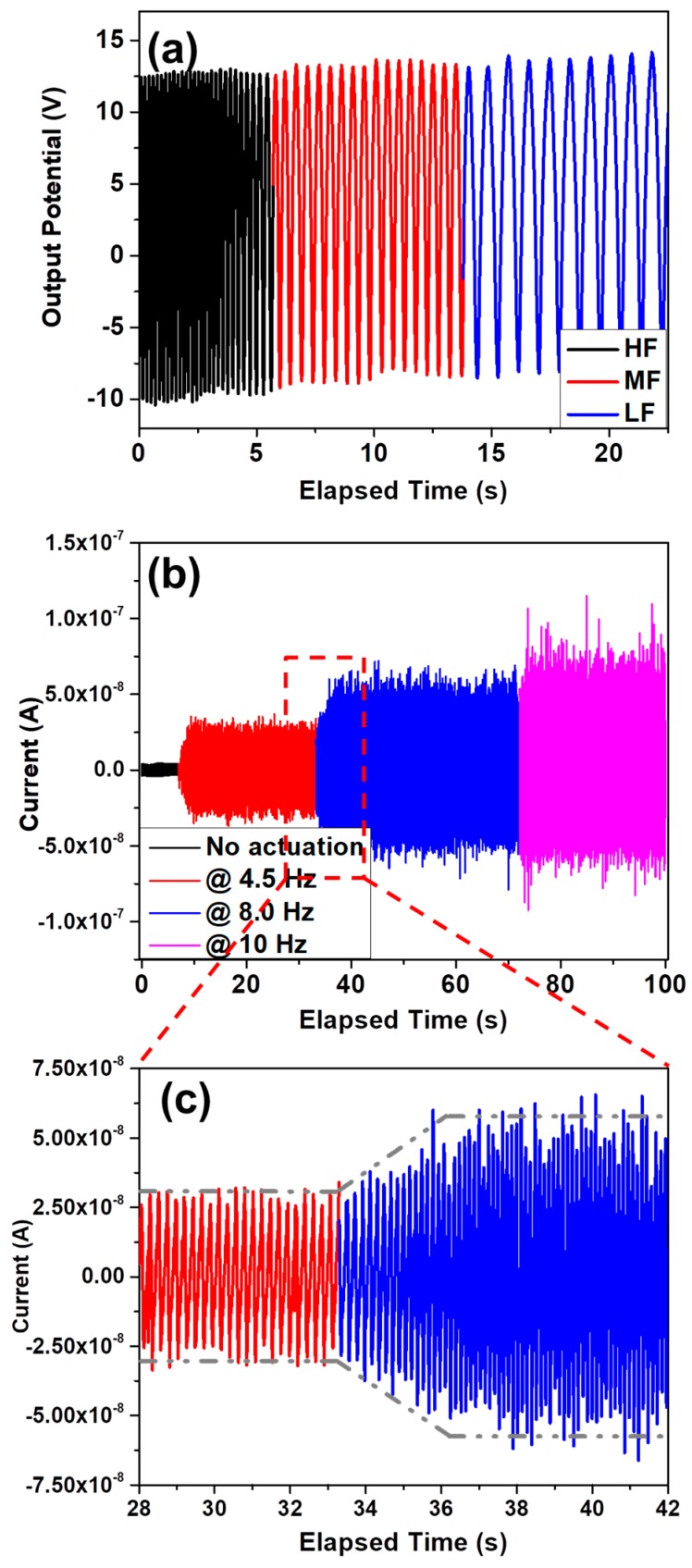
Piezoelectric output as a function of actuation frequency (**a**) open circuit voltages, high frequency (HF), medium frequency (MF), and low frequency (LF); (**b**) short circuit current; and, (**c**) the trend of output current by increasing the frequency of actuation source.

The piezoelectric performance of the device was evaluated after connecting the GaN NW-based PNG to potentiostat, while the device was actuated by a cyclic stretching-releasing agitation mechanism that was driven by a linear motor. At actuation frequency of 8 Hz, the open circuit output voltages and short circuit current were found 15.4 V and 64 nA, respectively, as shown in Figure 3a and 3b, respectively, while Figure 3c shows the change in piezoelectric current during the transition period of actuation source from on-state to off-state. Moreover, the effect of actuation frequency was comprehensively evaluated by varying the actuation frequency from 1 Hz to 10 Hz. The output voltages were constant throughout the actuation range with an optimum value of 13.6 V as depicted in Figure 4a. The actuation frequency was categorized as high frequency, medium frequency, and low frequency. However, a linear tendency was observed in output current by increasing the actuation frequency. Out of all the measurements, three scan ranges are provided in Figure 4b, with an actuation frequency of 4.5 Hz, 8.0 Hz, and 10 Hz, exhibiting an output current of 34 nA, 64 nA, and 85 nA, respectively. Moreover, Figure 4c shows the increase in piezoelectric output current upon increasing the actuation frequency from 4.5 Hz to 8.0 Hz. Moreover, a video clip is provided in the supplementary information, which displays the measurement procedure of piezoelectric output performance.

The work function of Ni metal is 5.22 eV, while the electron affinity of GaN is 4.53 eV. After the fabrication of the device, the Fermi level of GaN and metal are aligned, as a result, the Fermi level of all regions, including PDMS, aligned at the same level. The top surface of c-axis GaN NWs is the Ga-polar plane, thus under compressive stress, negative piezoelectric charges appear at the top of NWs towards c-axis direction, while positive charges appear at the bottom that is the—c-axis direction. The top and bottom ITO electrodes are not in direct contact, thus a capacitive coupling is achieved between ITO/Ni metal contact and GaN NWs through PDMS dielectric layer on the top side of PNG, further, the capacitive coupling is also formed between the bottom side ITO contact and GaN NWs. Moreover, NWs matrix was flipped and was then transferred, thus, the negative charges appear on top ITO contact of PNG and negative on bottom contact upon compressing.

**Figure 5:**
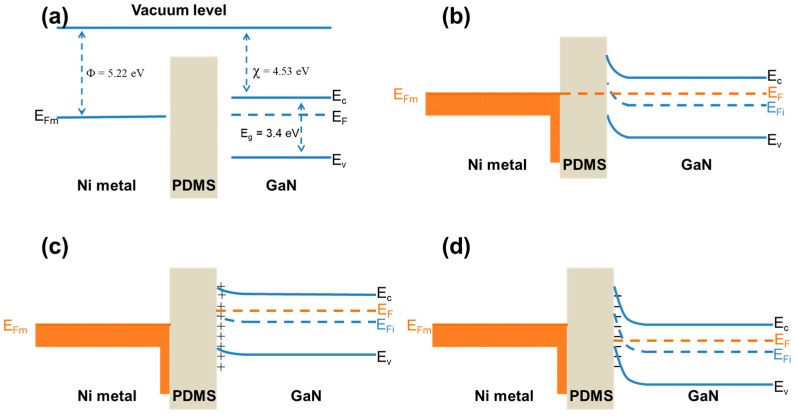
Schematic illustration of energy band diagram of Ni-PDMS-GaN (**a**) band positions of GaN and work function Ni in vacuum before contact; (**b**) metal-dielectric-semiconductor (MDS) interface in thermal equilibrium; (**c**) the effect of positive piezoelectric charges on band bending of GaN at PDMS-GaN interface; and, (**d**) formation of negative charges when the stress was relieved and their effect on band bending of GaN at PDMS-GaN interface. Φ—work function, χ—Electron affinity, E_F_—Fermi level, E_Fi_—Intrinsic Fermi level, E_g_—bandgap, E_c_—lower level of conduction band, E_v_—upper level of valance band, E_Fm_—metal fermi level.

The c-axis GaN NWs were intrinsically n-type, thus, due to the upward bending of valance band and the conduction band near surface of NWs, electrons are spatially separated from holes due to surface band bending [36,37]. Thus, the recombination of excited carriers is prohibited or nearly impossible due to the spatial separation. Therefore, the density of free carriers is reduced, resulting in higher RC time constant, which is the origin of suppressed free carrier screening. In order to enhance the suppression of free carrier screening, MDS interface was utilized. To understand the effect of MDS interface on free carrier screening a schematic illustration is given in Figure 5. The Figure 5a shows the band diagram of the materials before contact. When the materials were brought into contact then the Fermi level of complete structure aligned after thermal equilibrium, as depicted in Figure 5b. Under external compressive stress, positive piezoelectric charges appear at the GaN NW surface near MDS interface. Note that this GaN surface is N-polar c-plane because the NWs were flipped over during the lift-off process. As shown in Figure 5c, Fermi level in GaN is shifted to higher energy, which is the origin of the piezoelectric output potential. Due to the insulating nature of PDMS, further internal screening could be suppressed greatly and superior piezoelectric performance was achieved. When the external stress was removed, the piezoelectric charges disappear. But, the carriers redistributed due to the piezoelectric field still remain near the MDS interface and moves the Fermi level in the semiconductor downward (see Figure 5d). It explains why the polarity of NWs is reversed when the external stress on the PNG is released. Therefore, positive and negative peaks alternate under a series of stress pulses. 

The piezoelectric performance of this GaN NW array based PNG is compared with NW array based PNG that is composed of other material systems. The piezoelectric output of ZnO NW arrays was reported to be 20 V and 58 V in the literature [38,39], and that of lead zirconate titanate (PZT) NW array based PNG was reported up to 209 V [40]. Even though the piezoelectric output of our PNG is lower than ZnO and PZT NW based PNGs, but it is a reasonable output from GaN NWs because GaN is not researched intensively for piezoelectricity.

**Figure 6:**
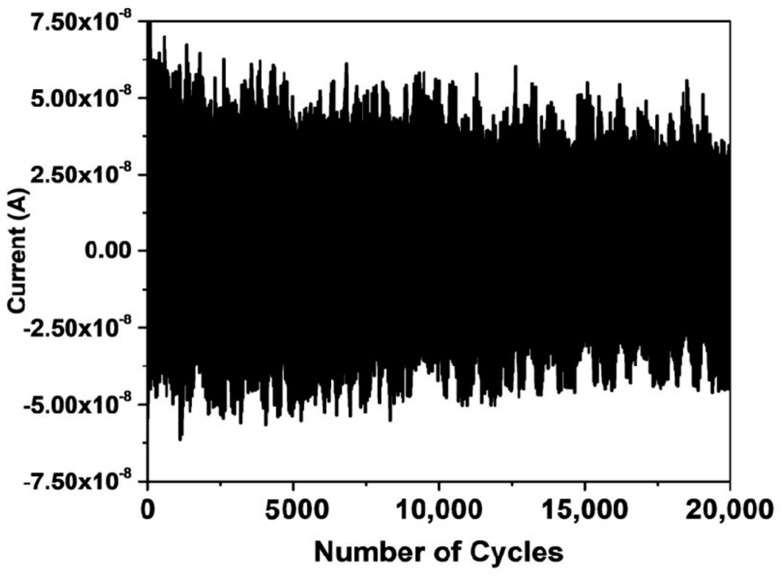
The long-term stability test of PNG with an actuation frequency of 8.0 Hz, piezoelectric output current for 20,000 cycles.

In order to evaluate the compatibility of our device in a real application, a long-term stability test was performed for 20,000 cycles under an actuation frequency of 8 Hz, as shown in Figure 6. The device was found rigorous and stable, an average piezoelectric output current reduced to 52 nA from 64 nA. Under the same degradation, the device is expected to be used for 60,000 cycles with the 50% degradation in its output. The underlying reasons for long-term stability of our device were the high aspect ratio and the flexibility of our device. Due to high aspect ratio, NWs can be bent very easily when compared to thin film counterparts. Such high piezoelectric bias, high stability, and high efficiency makes our design promising for a variety of applications in the sensors network. In the future, the same phenomena of MDS interface can be studied using co-axial configuration for very high output, which can be achieved due to the lateral suppression of free carrier screening along c-axis GaN NW based PNGs.

## 4. Conclusions

Well aligned c-axis GaN NWs were grown for the fabrication of flexible PNG. The transfer of the PDMS matrix embedded with c-axis GaN NWs on Si-rubber substrate was carried out using doctor’s blading, followed by the completion of device fabrication. The piezoelectric performance was measured, while the flexible PNG was actuated by a cyclic stretching-releasing agitation mechanism driven by a linear motor. A maximum open circuit output voltages and short circuit current of 15.4 V and 85 nA were measured, respectively. Moreover, the piezoelectric output of flexible PNG was evaluated as a function of actuation frequency. The actuation frequency was varied up to 10 Hz; piezoelectric output voltages remained constant throughout the range of actuation frequency, while a significant increase of piezoelectric current was observed by increasing the actuation frequency. The constant output voltages of 13.6 V were measured for all of the actuation range while the output current was found 34 nA, 64 nA, and 85 nA at actuation frequencies of 4.5 Hz, 8.0 Hz, and 10 Hz, respectively. Such high piezoelectric output was attributed to the surface Fermi-level pinning of GaN NWs, which reduced the free carriers inside GaN NWs, ultimately reducing free carriers screening. Moreover, the suppression of free carrier screening was enhanced due to the MDS interface, which inverts the surface of GaN NWs accordingly. At the end, the feasibility of our device for a real application was also checked by performing the long term stability test for 20,000 cycles, and the device performance was degraded less than 18% of its initial output. The design and the piezoelectric output make our device intriguing for a variety of applications in the field of sensors and ambient energy harvesting applications.

## Supplementary Materials

The following are available online at http://www.mdpi.com/2079-4991/8/6/437/s1, Video S1: Measurement of piezoelectric output current as a function of actuation frequency.
